# A Rare Case of Pneumonia Caused by Capnocytophaga canimorsus

**DOI:** 10.7759/cureus.66642

**Published:** 2024-08-11

**Authors:** Rabail Naseem, Izna Najam Syed, Waqar Hassan

**Affiliations:** 1 Medicine, Kettering General Hospital, Kettering, GBR; 2 General Surgery, The Royal Wolverhampton National Health Service (NHS) Trust, Wolverhampton, GBR; 3 Acute Medicine, Kettering General Hospital, Kettering, GBR

**Keywords:** pneumonitis, outcome, epidemiology, clinical features, capnocytophaga canimorsus

## Abstract

We present the case of a 59-year-old immunocompetent female with a mild cough, fever, and rash. She was diagnosed with mild pneumonitis caused by *Capnocytophaga canimorsus*, with no history of dog bites. An indolent clinical course with transmission via canine face licking in immunocompetent individuals is a rare occurrence according to the literature. The diagnosis was made on positive blood cultures and polymerase chain reaction, following which the patient was treated with beta-lactam antibiotics. *C. canimorsus* is a gram-negative bacterium found in the saliva of dogs and cats. The incidence of human infections is rare, particularly affecting immunocompromised patients exposed to the saliva of these animals. Typical manifestations include severe sepsis, with a high case fatality.

## Introduction

*Capnocytophaga canimorsus* are anaerobic gram-negative bacteria, which are normal commensals of dog and cat saliva [1]. *C. canimorsus* may be responsible for significant human infections following its transmission, mainly via dog bites [2]. However, some transmission modes include scratches, licks, and, in some cases, mere exposure to host animals [3]. Immunocompromised individuals, such as those with asplenia or hyposplenism, excessive alcohol consumption, or immunosuppressive treatment are particularly vulnerable to *C. canimorsus* infection [4]

*C. canimorsus* is one of the rarer organisms associated with dog bites. Typical clinical manifestations of *C. canimorsus* infection include sepsis, disseminated intravascular coagulation, endocarditis, meningitis, and gangrenous necrosis of the digits [5,6]. Infection with *C. canimorsus* can be fatal, especially in the setting of sepsis in elderly or immunocompromised patients [6]. Therefore, it is clear that *C. canimorsus* infection rarely follows an indolent course, with limited cutaneous and pulmonary manifestations [7], as seen in our patient. There have been a few reported cases of non-severe *C. canimorsus* and *C. canimorsus*-like infections, presenting as fever, with pneumonia-like illnesses, particularly in immunocompromised patients [8,9]. Diagnosis is made on positive blood cultures and polymerase chain reaction (PCR) [6]. Therefore, it is important to request blood cultures and PCR in patients presenting with pyrexia and a history of canine or, less commonly, feline exposure [2].

Here, we present a case of *C. canimorsus* infection with an indolent clinical course, fever, rash, and mild pneumonitis. To our knowledge, similar clinical presentations have not been described previously.

## Case presentation

A 59-year-old female attended the accident and emergency department with a one-day history of pyrexia, dry cough, and a non-blanching rash on bilateral lower limbs. The patient had been fit and well until the day of her presentation to the emergency department. Her medical history was significant for haemorrhoids, menorrhagia, and depression.

**Figure 1:**
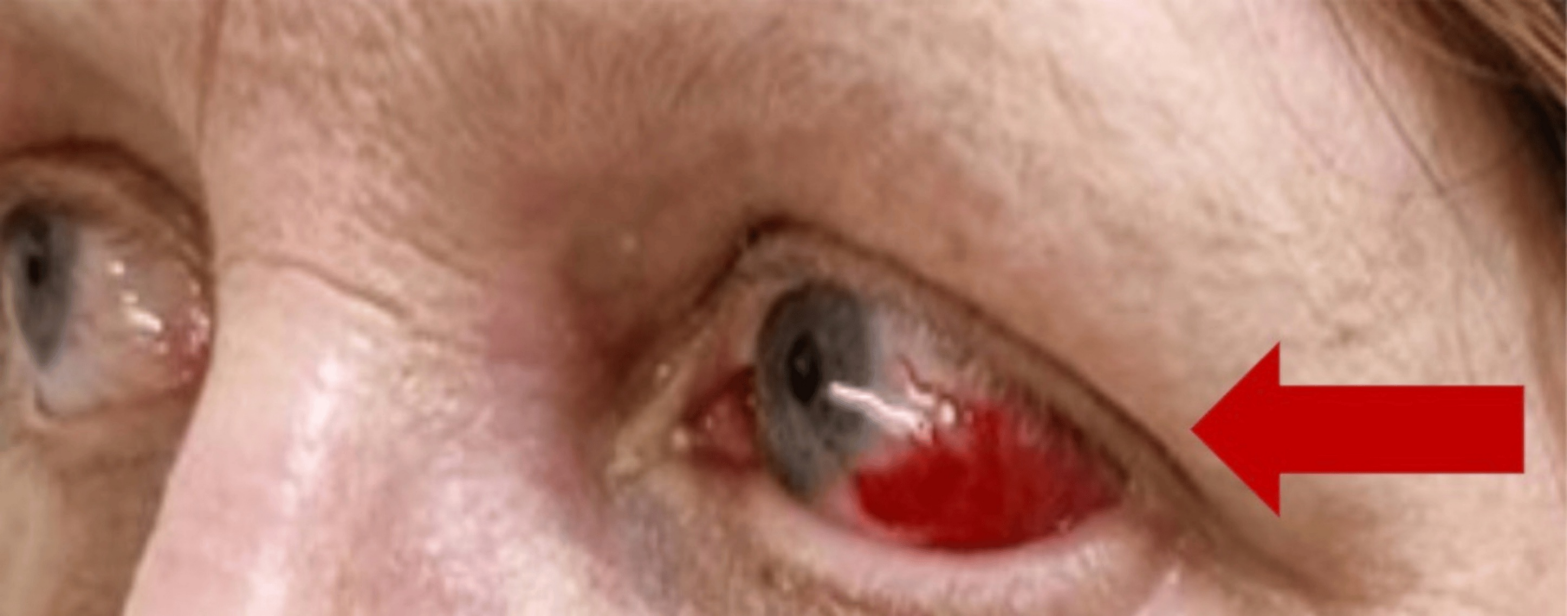
Left subconjunctival haemorrhage.

**Figure 2:**
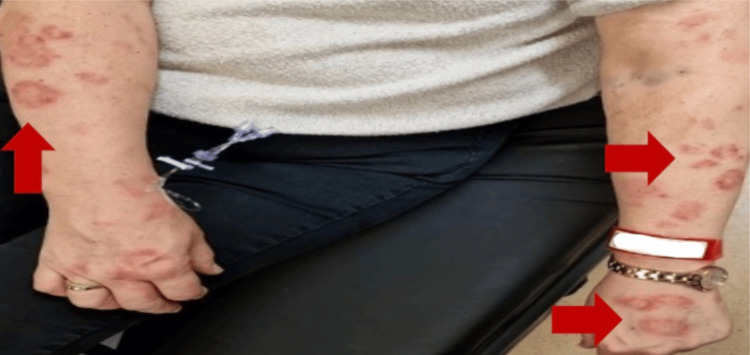
Target lesions on upper extremities.

**Figure 3:**
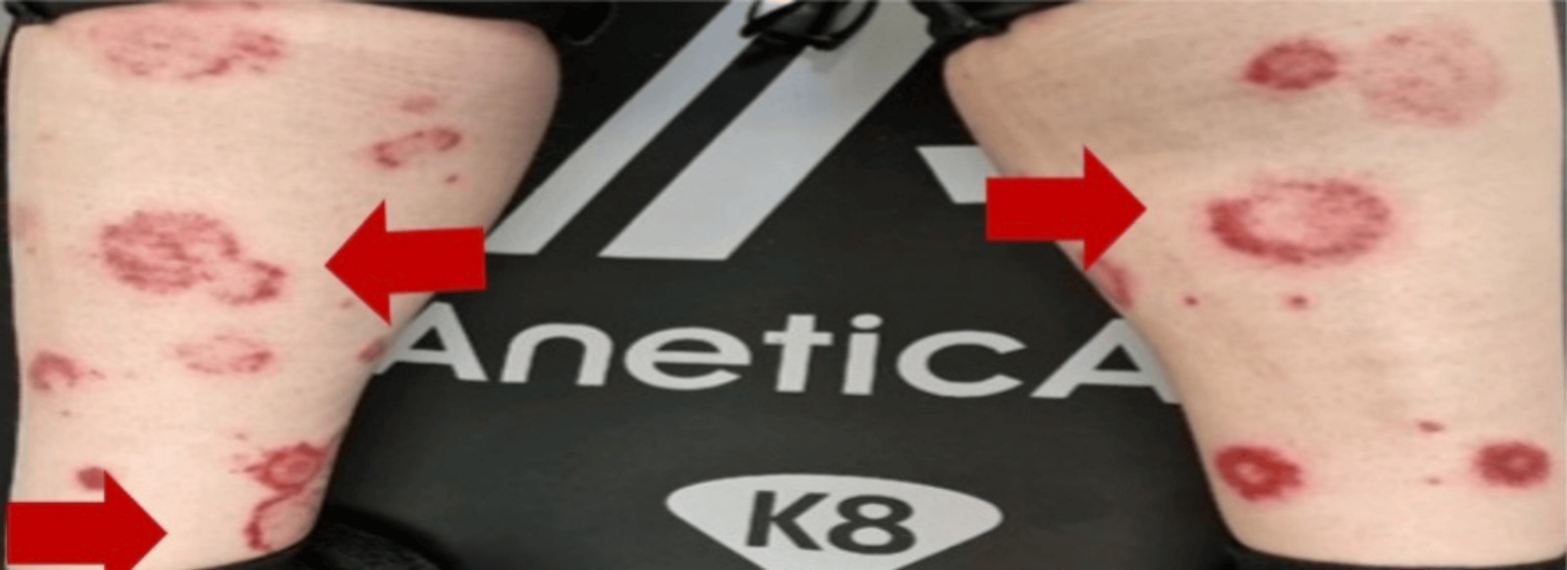
Target lesions on lower extremities.

On physical examination, the patient was hemodynamically stable, with a temperature of 38.5°C. She had a subconjunctival haemorrhage in the left eye (Figure 1), as well as a few oral ulcers. Multiple target lesions (areas of normal skin surrounded by areas of erythema) were found on bilateral upper and lower limbs (Figures 2, 3). The lesions were non-blanching and non-pruritic.

Her laboratory parameters showed a markedly raised C-reactive protein (Table 1).

**Table 1:** Laboratory parameters.

Laboratory parameters	Reference range	Day 1	Day 2	Day 4	Day 14
C-reactive protein	<5 mg/dL	271	281	201	6
Total leucocyte count	4-11 × 10^9 ^cells	9 × 10^9^	9.2 × 10^9^	5.8 × 10^9^	5.6 × 10^9^
Procalcitonin	0.00–0.25 ng/mL	3.21	-	-	-

**Figure 4:**
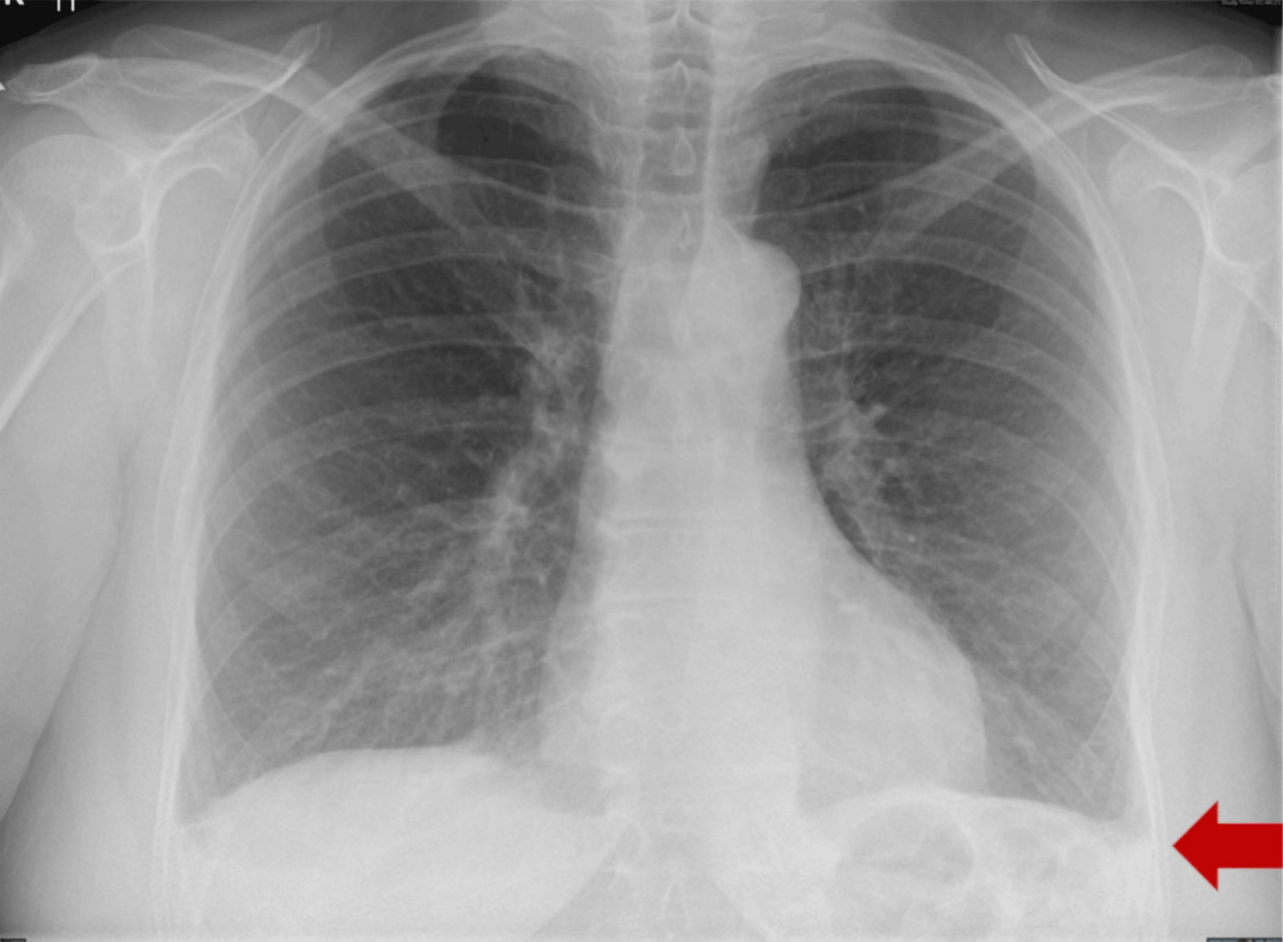
Chest X-ray posteroanterior view showing a small left-sided pleural effusion (red arrow).

A chest X-ray showed a small pleural effusion bilaterally (Figure 4).

**Figure 5:**
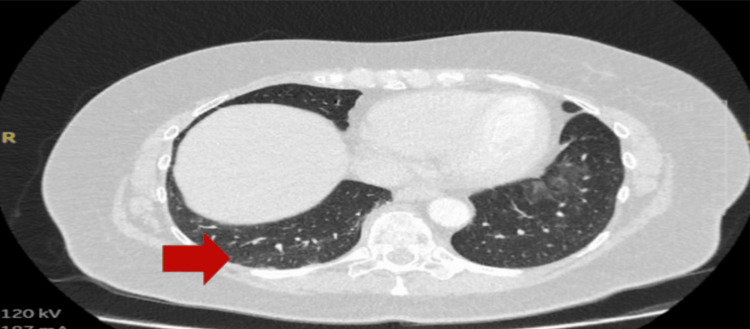
High-resolution CT of the chest showing subtle right middle lobe ground-glass changes (red arrow).

The next day, the patient developed a headache and worsening temperature spikes. However, on examination, there was no neck stiffness, photophobia, or focal neurological deficits. She underwent a CT of the head, which did not show any abnormalities. She underwent a high-resolution CT (HRCT) of the chest on account of a history of dry cough with fever and rising inflammatory markers (Table 1). The HRCT showed focal right middle lobe ground-glass opacification, likely inflammatory or infectious (Figure 5).

The patient was suspected to have community-acquired pneumonia and cutaneous systemic lupus erythematosus versus erythema multiforme secondary to viral pneumonitis. Topical steroids were initiated, with continuation of IV antibiotics, and the patient demonstrated clinical (resolving rash and fever) and biochemical improvement (Table 1). She was reviewed by ophthalmology for her subconjunctival haemorrhage, with reassurance of self-resolution of the haemorrhage. Over the next four days, the patient’s autoimmune profile and complement levels were assessed (Table 2).

**Table 2:** Autoimmune profile and complement levels.

Laboratory parameters	Reference range	Patient’s results
Anti-nuclear antibodies	-	Negative
Immunoglobulins - IgG	-	Negative
Immunoglobulins - IgM	-	Negative
Immunoglobulins - IgA	-	Negative
C3	0.9–1.8	1.82
C4	0.14–0.54	1.43

Blood culture was positive was gram-negative bacilli. The blood culture did not yield any atypical organisms (*Legionella*, pneumococci, Epstein-Barr virus, cytomegalovirus). The patient continued to improve clinically and was deemed well enough to be discharged on oral clarithromycin as per advice from microbiology.

**Figure 6:**
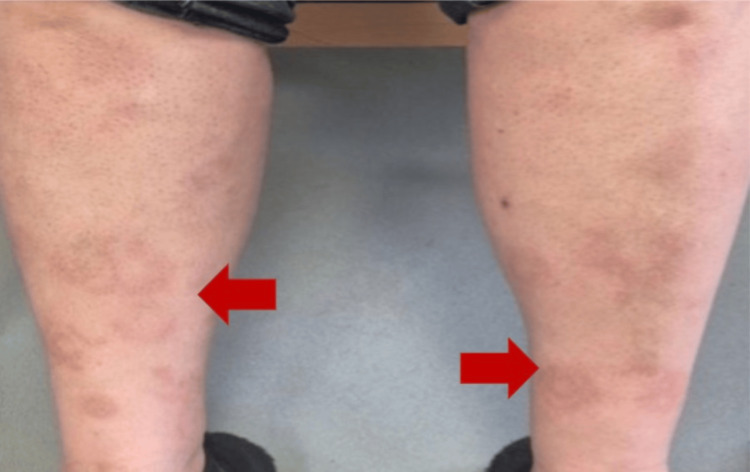
Resolving target lesions on the lower extremities.

Fourteen days later, the PCR and final blood culture report showed the presence of *C. canimorus* on blood agar. Microbiology was consulted and advised that the patient be treated with intravenous clarithromycin, as well as for the patient to undergo an echocardiogram to rule out infective endocarditis. The patient was readmitted to the hospital for inpatient management. However, she was clinically well at this presentation, with a resolution of previous symptoms, including the previous rash (Figure 6).

The patient denied any recent dog bites. However, she admitted that she is a dog carer, and is habitual of kissing her puppy on the mouth. On physical examination, the patient did not have any evidence of current or previous open wounds, new murmurs, or any peripheral stigmata of infective endocarditis. On this occasion, laboratory investigations did not yield any significantly raised markers. She did not have any valvular abnormalities or features of infective endocarditis on the echocardiogram. Following a 14-day course of clarithromycin, the patient made a full recovery.

## Discussion

*Capnocytophaga* is a slow-growing, capnophilic, anaerobic gram-negative, oxidase, and catalase-positive bacterium [10]. *Capnocytophaga* species, i.e., *C. canimorsus* and *C. cynodegmi*, are commonly found in the canine and feline oral cavities [11].

In the United Kingdom, around 6,000 people are hospitalised annually due to canine bites [12]. Rabies, *Pasteurella*, and *Bartonella* are among the organisms associated with dog bites [6]. Human infections with *C. canimorsus* are rare at 0.67 per million population [5]. *C. canimorsus* is not a notifiable disease in the United Kingdom. Hence, only provisional national data exists on the incidence of *C. canimorsus* infections. In 2022, 10 cases of *C. canimorsus* and one case of *C. ochracae* were reported in the United Kingdom, of which six were males and five were females [13]. Existing literature reports most cases in males, with a median age of 58 years [10].

Three unique factors were identified in the case under discussion. The first observation was the immunocompetent status of the patient as well as the indolent nature of *C. canimorsus* infection in the patient. *C. canimorsus* infection is known to occur in the setting of immunocompromise in patients with older age, alcoholism, prior splenectomy, immunosuppressive medication, diabetes mellitus, hematopoietic stem cell transplantation, active malignancy, end-stage renal disease (ESRD), solid organ transplant, and human immunodeficiency virus (HIV) infection [11,14,15]. Our patient, although within the vulnerable age group, was immunocompetent. She did not have a history of diabetes, stem cell or organ transplant, ESRD, or HIV infection. She did not consume excess alcohol, her blood tests showed no features of immunocompromise, and her spleen appeared morphologically normal. This may be indicative of the reason for an atypical non-severe clinical course of *C. canimorsus* infection in the patient. A retrospective multicentre cohort study conducted in the United States between 2010 and 2020 examined the characteristics of patients with *Capnocytophaga* infection. Clinical presentations of *C. canimorsus* infection included sepsis (32%), fever of unknown origin (13%), meningitis (13%), cellulitis (11%), septic shock (9%), respiratory tract infection/inflammation (7%), phlebitis (2%), endocarditis (2%), urosepsis (2%), septic arthritis of knee joints (2%), diverticulitis (2%), and meningitis (2%) [10]. Although *C. canimorsus* infection has been associated with ophthalmic infections such as keratitis [16] and conjunctivitis [11], conjunctival haemorrhages have been described in the presence of disseminated intervascular coagulopathy [17]. This was not found in our patient, who presented solely with a fever and rash. Although initially suspected to have an autoimmune condition, the patient continued to have fever spikes, as a result of which the blood cultures were sent. Therefore, it is important to consider *C. canimorsus* as a causative agent in cases of pyrexia with no obvious cause, and a detailed history and examination should be undertaken, including the history of canine exposure, and any open wounds (subtle or obvious).

Isolates of *C. canimorsus* can be detected in blood, cerebrospinal fluid, and respiratory tract [10]. Diagnosis of the organism on cultures can be challenging due to its slow growth [18]. Given that blood cultures can initially be negative (up to six days), extended incubation times should be taken into account. Moreover, 16 small ribonucleic acid sequencing and PCRs are beneficial in identifying *C. canimorsus* isolates [19,20]. Blood cultures and PCR are often taken for patients requiring hospitalisation. Therefore, in less severe cases that do not require hospitalisation, blood cultures may not be requested. In our patient, the diagnosis was made on positive blood cultures and PCR which were taken a few days after admission. As in this case, the exposure to dog saliva was discovered later, emphasising the need to inquire about contact with animals when diagnosing patients with unexplained fevers. If there is a history of animal contact, even in the absence of animal bites, blood cultures should be requested, followed by PCR if not positive and other diagnoses are unlikely.

The third anomaly in this case of *C. canimorsus* infection was the absence of a history of canine bites. Historically, animal-to-human transmission occurs via animal bites, when an open wound comes into contact with animal saliva [11,14]. The average incubation period is three days or less from exposure to animal saliva [7,11,14], with shorter incubation periods in cases with higher infectious doses [11,14]. Hence, it is important to consider *C. canimorsus* as a plausible diagnosis in case of animal exposure, even in the absence of dog bites or open wounds with exposure to canine or feline saliva.

Penicillin and other beta-lactam drugs are highly effective against *C. canimorsus* due to the rare prevalence of beta-lactamase [21]. Literature reviews have described the effectiveness of clindamycin, doxycycline, amoxicillin/clavulanic acid, and fluoroquinolones in treating mild *C. canimorsus* infections. Carbapenems have been demonstrated to be beneficial in treating soft-tissue infections and multidrug-resistant strains. The duration of the treatment is not clear, with a vast majority of cases showing clinical improvement after seven to ten days of treatment. Additionally, some case reports have recommended the administration of prophylactic amoxicillin/clavulanic acid after dog bites [11,14,15,22]. No data exists on the role of macrolides in treating *C. canimorsus* infections. Our patient was treated with meropenem and clarithromycin with excellent clinical outcomes.

Although cases of *C. canimorsus* infection with non-severe bacteremia exist, they are likely underreported due to publication bias [6]. The case fatality of *C. canimorsus* infections has been reported to be 30% [10]. However, considering the reporting bias for non-severe cases, coupled with the lack of understanding of the clinical features of *C. canimorsus* infection and the fact that *C. canimorsus* is not a notifiable disease, it is possible that the mortality rate is not as high as it appears. This case of uncomplicated bacteremia in an immunocompetent patient may provide the foundation for a better understanding of the clinical course of *C. canimorsus*. Furthermore, a surveillance and reporting system for *C. canimorsus* infection needs to be established to get more accurate statistics regarding morbidity and case fatality.

## Conclusions

*C. canimorsus* infections are more commonly seen in immunocompromised patients. However, our patient was not immunosuppressed. The usual mode of transmission is due to dog bites; however, in our patient, there was no history of a dog bite. The likely transmission was via direct contact of the dog saliva with the patient’s oral mucosa following the dog licking the patient’s face. An indolent clinical course with mild respiratory tract infection and cutaneous manifestations is rare. Cases are underreported and undiagnosed due to the slow growth of bacteria on culture media. Most cases of *C. canimorsus* infection respond well to antimicrobial treatment. We aim to highlight the unusual mode of transmission, clinical manifestations, and treatment response in our patient with *C. canimorsus* infection. This article will provide scope for further studies on the aetiology, pathogenesis, and treatment options for patients with *C. canimorsus* infection.
